# Loss of miR-638 *in vitro* promotes cell invasion and a mesenchymal-like transition by influencing SOX2 expression in colorectal carcinoma cells

**DOI:** 10.1186/1476-4598-13-118

**Published:** 2014-05-23

**Authors:** Kelong Ma, Xiaorong Pan, Pingsheng Fan, Yinghua He, Jun Gu, Wei Wang, Tengyue Zhang, Zonghai Li, Xiaoying Luo

**Affiliations:** 1 State Key Laboratory of Oncogenes & Related Genes, Shanghai Cancer Institute, Renji Hospital, Shanghai Jiaotong University School of Medicine, No. 25/Ln2200, XieTu Rd, Shanghai 200032, China; 2 School of Life Science, Anhui Medical University, Hefei 230032, China; 3 School of Integrated Western and Chinese Medicine, Anhui University of Chinese Medicine, Hefei 230038, China; 4 Anhui Provincial Cancer Hospital, Anhui Medical University, Hefei 230031, China

**Keywords:** miR-638, SOX2, CRC, Invasion

## Abstract

**Background:**

Colorectal carcinoma (CRC) is a major cause of cancer mortality. The aberrant expression of several microRNAs is associated with CRC progression; however, the molecular mechanisms underlying this phenomenon are unclear.

**Methods:**

miR-638 and SRY-box 2 (SOX2) expression levels were detected in 36 tumor samples and their adjacent, non-tumor tissues from patients with CRC, as well as in 4 CRC cell lines, using real-time quantitative RT-PCR (qRT-PCR). SOX2 expression levels were detected in 90 tumor samples and their adjacent tissue using immunohistochemistry. Luciferase reporter and Western blot assays were used to validate SOX2 as a target gene of miR-638. The regulation of SOX2 expression by miR-638 was assessed using qRT-PCR and Western blot assays, and the effects of exogenous miR-638 and SOX2 on cell invasion and migration were evaluated *in vitro* using the HCT-116 and SW1116 CRC cell lines.

**Results:**

We found that miR-638 expression was differentially impaired in CRC specimens and dependent on tumor grade. The inhibition of miR-638 by an antagomiR promoted cell invasion and a mesenchymal-like transition (lamellipodium stretching increased and cell-cell contacts decreased, which was accompanied by the suppression of the epithelial cell marker ZO-1/E-cadherin and the upregulation of the mesenchymal cell marker vimentin). A reporter assay revealed that miR-638 repressed the luciferase activity of a reporter gene coupled to the 3′-untranslated region of SOX2. miR-638 overexpression downregulated SOX2 expression, and miR-638 inhibition upregulated SOX2 expression. Moreover, miR-638 expression levels were correlated inversely with SOX2 mRNA levels in human CRC tissues. The RNAi-mediated knockdown of SOX2 phenocopied the invasion-inhibiting effect of miR-638; furthermore, SOX2 overexpression blocked the miR-638-induced CRC cell transition to epithelial-like cells.

**Conclusions:**

These results demonstrate that the loss of miR-638 promotes invasion and a mesenchymal-like transition by directly targeting SOX2 *in vitro*. These findings define miR-638 as a new, invasion-associated tumor suppressor of CRC.

## Background

MicroRNAs (miRNAs) play pivotal roles in physiological and pathological processes via their regulation of a wide variety of genes, predominantly through their interaction with the 3′-untranslated regions (3**′**UTR) of their corresponding mRNA targets [1,2]. More than 4,665 mature miRNA products have been annotated in the human genome, according to the most recent version of the miRBase program (Release 20: June 2013; http://www.mirbase.org/), and increasing evidence has shown that the deregulation of miRNAs is involved in the pathogenesis of a wide range of diseases, such as human cancers [3,4]. However, the roles of most miRNAs in tumor initiation and progression are still unknown.

Colorectal carcinoma (CRC) is the fourth-most common cause of cancer-related mortality worldwide [5]. Approximately 715,000 deaths from CRC are estimated to occur annually, accounting for 8% of all cancer deaths [6]. Because of advancements in CRC treatment regimens, there has been substantial progress in the treatment for colorectal cancer, and survival rates have improved over the past 40 years [7]. Metastasis is the major concern in cancer therapy; cell invasion and the epithelial-to-mesenchymal transition (EMT) is the primary step in this process.

The EMT is a biological process in which a polarized epithelial cell, which normally interacts with the basement membrane via its basal surface, undergoes multiple biochemical changes that cause the epithelial cell to assume a mesenchymal cell phenotype. These phenotypic changes include enhanced migratory capacity, invasiveness, elevated resistance to apoptosis, and a greatly increased production of ECM components [8]. Mounting evidence suggests that the EMT occurs in CRC [9,10]. Recent studies have revealed that miRNAs are involved in the EMT process in CRC cells; for example, miR-101 [11], miR-212 [12], miR-155 [13], miR-130b [14], and miR-34 [15] have been found to be involved in the EMT process in CRC cells. One study has provided evidence that miR-638 is downregulated at the invasive front of CRC [16]; however, its expression and function were not addressed.

In the present study, we sought to determine the role of miR-638 in CRC progression. We defined miR-638 as a new, invasion-associated tumor suppressor miRNA *in vitro*. Moreover, we identified SOX2, a factor that can induce pluripotent stem cells [17], as a direct, functional target of miR-638.

## Materials and methods

### Patients and tissue microarray

Participants who provided samples also provided written, informed consent to participate in this study. The Ethics Committee of the Shanghai Cancer Institute approved the study, the consent procedure, and the tissue array study. All of the research was performed in China. Paired colorectal tumor tissues and their corresponding adjacent non-tumor colorectal tissues (5 cm away from the lesions) were collected from patients who underwent curative surgery for CRC at Anhui Medical University, Anhui Province, China. Normal colon tissue was collected from patients with non-cancerous colon disease. A CRC diagnosis was confirmed by histological examination, and the relevant clinical and pathological information was retrieved from the hospital database (Additional file 1: Table S1a). Glass-slide tissue arrays for CRC were purchased from the Shanghai Outdo Biotech Co., Ltd. (Shanghai, China) (Additional file 1: Table S2a), and immunostaining (SOX2, ab75485, 1:100, Abcam, Cambridge, MA; vimentin, #5741, 1:50, Cell Signaling Technology, Beverly, MA) was performed on the tissue microarray slides. Staining was analyzed based on the percentage of positively stained cells and staining intensity by a pathologist or using Image-Pro Plus 6.0 software (Media Cybernetics, Inc., Bethesda, MD) (Additional file 2).

### Cell culture

Four human CRC cell lines were purchased from the American Type Culture Collection (ATCC, Rockville, MD, USA). HCT-116 cells (ATCC No. CCL-247) were maintained in McCoy’s 5A medium, LoVo cells (ATCC No. CCL-229) were maintained in F-12 K medium (Kaighn’s Modification of Ham’s F-12 Medium), and SW480 cells (ATCC No. CCL-228) and SW1116 cells (ATCC No. CCL-233) were maintained in Leibovitz’s l-15 medium. The media were supplemented with 10% fetal bovine serum, and the cells were incubated in a humidified atmosphere of 95% air and 5% CO_2_ at 37°C.

### Transfections

miRNA mimics and miRNA antagomiRs were designed and synthesized by RiboBio (Guangzhou, China). The miRNA antagomiRs were composed of nucleotides with a 2′-O-methylmodification. The SOX2 siRNAs (sense, 5′-GGAAUGGACCUUGUAUAGAUC-3′; anti-sense, 5′-UCUAUACAAGGUCCAUUCCCC-3′) were synthesized by RiboBio (Guangzhou, China), and the SOX2 overexpression construct was obtained from Origene Inc. (Beijing, China). The miRNA mimics, miRNA antagomiRs, SOX2-targeted siRNA, and SOX2 overexpression construct were transiently co-transfected with GFP (transfection efficiency control) using an Amaxa Nucleofector (Amaxa, Koeln, Germany) according to the manufacturer’s protocol.

### RNA extraction and quantitative real-time RT-PCR (qRT-PCR)

Total RNA was extracted using TRIzol reagent (Invitrogen, Carlsbad, CA) according to the manufacturer’s protocol. qRT-PCR with miRNA was performed using the TaqMan Reverse Transcription Kit (Applied Biosystems), TaqMan MicroRNA Assays (Applied Biosystems), and a LightCycler TaqMan Master (Roche Diagnostics, Mannheim, Germany) according to the manufacturers’ instructions. The miRNA expression levels were calculated using the delta-delta Ct method with RNU6B as an internal control. A Ct value of 35 was set as the cut-off value for defining as non-detected.

cDNA was reverse-transcribed from 1 μg of RNA using the SYBR®Prime Script™ RT-PCR kit (Takara Biochemicals, Tokyo, Japan), and the reactions were performed on an ABI PRISM®7900HT Real-Time PCR System. The thermal cycling conditions were as follows: an initial step at 95°C for 15 s followed by 40 cycles of 95°C for 5 s and 60°C for 30 s. Each experiment was performed in a 20-μl reaction volume containing 10 μl of SYBR® Prime Ex Taq™ II (2×), 0.8 μl of forward primer and reverse primer (10 μM each), 0.4 μl of ROX Reference Dye or Dye II (50×), 2 μl of cDNA, and 6 μl of H_2_O. β-Actin was used as an internal control. The quantification of the mRNA was calculated using the comparative Ct (the threshold cycle) method according to the following formula: Ratio = 2^-*ΔΔ*ct^ = 2^- [*Δ*Ct(sample) - *Δ*Ct(calibrator)]^, where ΔCt is equal to the Ct of the target gene minus the Ct of the endogenous control gene (β-actin). The primers for SOX2 (SOX2RTF: 5′-CGAGATAAACATGGCAATCAAAAT-3′; SOX2RTR: 5′-AATTCAGCAAGAAGCCTCTCCTT-3′) have been described previously [18]. The internal control actin primers were designed as previously described [19,20].

### Western blot analysis

Proteins were separated by SDS-PAGE and transferred to a polyvinylidene fluoride membrane (Bio-Rad, Hercules, CA). The membrane was blocked with 5% non-fat milk and incubated with the following antibodies: the epithelial cell marker ZO-1 (1:200) and E-cadherin (1:1000), the mesenchymal cell marker vimentin (1:50,000), SOX2 (1:2,000), Myc (1:2,000), β-actin (1:2,000, Santa Cruz Biotechnology, Santa Cruz, CA), and GAPDH (1:10,000; Kang-Chen Bio-tech Shanghai, China). And the quantification of Western Blot exerted by ImagineJ software (NIH, USA).

### Cell migration and invasion assays

We transfected the miR-638 mimics and SOX2 siRNA into HCT-116 and SW1116 cells using an Amaxa Nucleofector (Amaxa, Koeln, Germany). Cell migration and invasion were evaluated using Matrigel-uncoated and -coated transwell chambers (cat. 3422, Corning, NY). Briefly, 5 × 10^4^ cells were suspended in 200 μl of DMEM without serum and placed into cell culture inserts (8-μm pore size; BD Falcon, San Jose, CA) of a companion plate (BD Falcon San Jose, CA) in pre-warmed culture medium containing 10% fetal bovine serum in the well. The cells were incubated overnight at 37°C in 5% CO_2_ and then fixed in 4% paraformaldehyde in PBS. Cell migration and invasion were determined by staining the cells with 0.1% crystal violet (Sigma, St Louis, MO) and counting the cells under a light microscope (100× magnification) in eight randomly selected areas.

### Luciferase reporter assay

Luciferase activity was detected using the Dual Luciferase Assay (Promega, USA) according to the manufacturer’s protocols. The transfected cells were lysed in tissue culture dishes with lysis buffer, and the lysates were centrifuged at maximum speed for 1 min in an Eppendorf microcentrifuge. The relative luciferase activity was determined using a Modulus TD20/20 Luminometer (Turner Biosystems, Sunnyvale, CA), and the transfection efficiency was normalized to *Renilla* activity.

### Immunofluorescence imaging

Transfected SW1116 cells were seeded at a density of 2 × 10^4^ onto poly-L-lysine-coated glass coverslips in a 6-well plate. After further culture overnight, the cells were permeabilized with 0.1% Triton X-100 (Sigma-Aldrich, St. Louis, MO). For filamentous actin (F-actin) staining, the coverslips were incubated with TRITC-labeled phalloidin (Sigma-Aldrich, St. Louis, MO) at room temperature, and the cell nuclei were counterstained with DAPI. The cells were co-transfected with 40 ng of pEGFP plasmid as a control.

### Statistical analyses

All experiments were performed in triplicate. The data are presented as the mean values ± standard error of the mean (SEM) and were analyzed using Student’s *t*-test. *p* values less than 0.05 were considered significant. Statistical analyses were performed using GraphPad Prism 5.01 software (GraphPad Software Inc., San Diego, CA).

The accession numbers for miR-638 is MIMAT0003308, and that for SOX2 is NM_003106.2.

## Results

### miR-638 shows reduced expression in colorectal carcinoma

**Figure 1:**
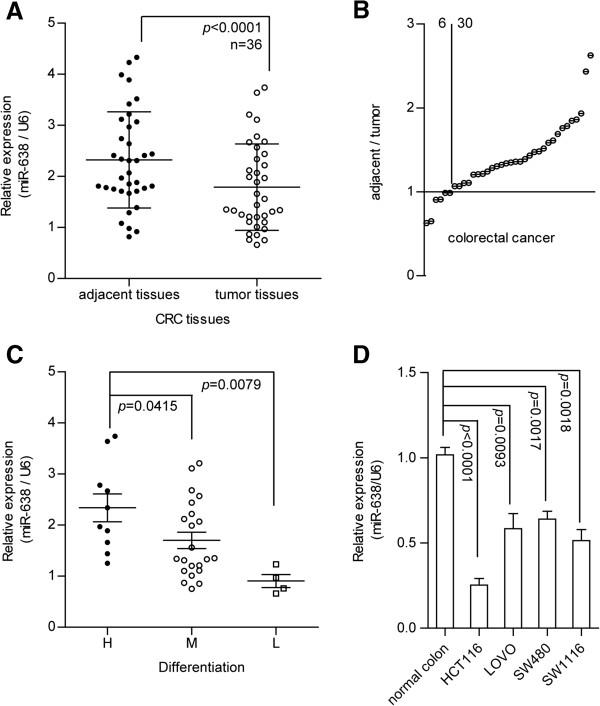
**miR-638 exhibits reduced expression in CRC tissues. A)** We analyzed the expression levels of miR-638 in 36 pairs of CRC tissues and observed a 22.98% decrease in expression in the CRC tissue samples compared with adjacent noncancerous tissue samples, *p* < 0.0001 (paired *t*-test). **B)** miR-638 expression was downregulated in 83.33% of the 36 pairs of tissues. **C)** miR-638 expression was correlated with tumor differentiation, and the miR-638 expression level was downregulated to 27.28% in moderately differentiated samples and to 61.29% in poorly differentiated samples compared with its levels in well-differentiated samples. H, M, and L indicate high, moderate, and low differentiation grades, respectively. **D)** miR-638 expression was reduced in CRC cell lines compared with normal colon tissue samples.

Previous microarray analyses revealed that 23 miRNAs are downregulated in CRC tissues (Additional file 1: Table S3), including miR-497 [21], miR-9 [22], miR-30a [23], and miR-139 [24]. To further screen miRNAs that are deregulated in CRC, qRT-PCR assays were conducted to evaluate the expression levels of these miRNAs in 36 pairs of CRC clinical samples. In addition to the four miRNAs described above, miR-638 was markedly downregulated in CRC tissues. The expression levels of miR-638 were decreased in 83.33% the samples (30/36; Figure 1B, Additional file 3: Table S1b) and a 22.98% decrease in expression in the CRC tissue samples compared with adjacent noncancerous tissue samples (2.323 to 1.789, p < 0.0001; Figure 1A). And a 27.28% decrease in moderately differentiated samples and 61.29% decrease in poorly differentiated samples compared to well-differentiated samples (Figure 1C). The miR-638 levels in all four CRC cell lines (HCT116, LoVo, SW1116, and SW480) were downregulated compared with that of normal colorectal tissues (Figure 1D). These results demonstrate that miR-638 showed reduced expression levels in CRC and was inversely correlated with tumor differentiation.

### miR-638 suppresses cell invasion and migration

**Figure 2:**
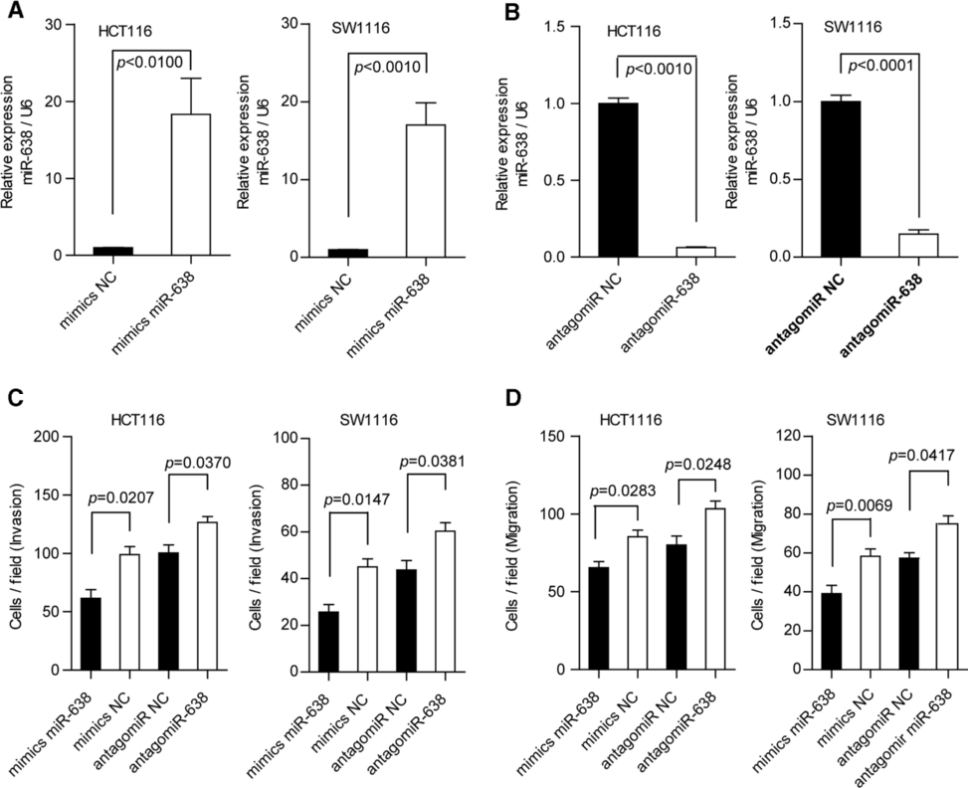
**miR-638 overexpression inhibits cell invasion and migration. (A)** miR-638 expression levels were confirmed by a qRT-PCR assay when miR-638 mimics were transfected into HCT116 and SW1116 cells. **(B)** miR-638 expression levels were confirmed by a qRT-PCR assay when antagomiR-638 was transfected into HCT116 and SW1116 cells. **(C)** Transfected miR-638 mimics suppressed cell invasion and transfected antagomiR-638 promoted cell invasion in HCT116 and SW1116 cells. **(D)** Transfected miR-638 mimics decreased cell migration, whereas transfected antagomiR-638 increased cell migration in HCT116 and SW1116 cells.

To understand the biological effect of miR-638 deregulation on the development of colorectal carcinoma, gain- or loss-of-function analyses were performed using an overexpression or silencing strategy through the transfection of miR-638 mimics or antagomiRs (using an Amaxa Nucleofector device) into the CRC cell lines HCT-116 and SW1116 (the miR-638 levels in the CRC cells were confirmed through qRT-PCR; Figure 2A and 2B). miR-638 elicited an apparent effect on the cell motility of CRC cells. Matrigel-coated (for invasion) and -uncoated (for migration) transwell assays were used to determine the invasiveness and migration of HCT-116 and SW1116 cells after transfection with miR-638 mimics or antagomiR-638. miR-638 overexpression reduced the number of invasive HCT-116 and SW1116 cells by 37.6% and 43.0%, respectively; however, antagomiR-638 enhanced cell invasion up to 20.8% and 27.6%, respectively (Figure 2C, Additional file 4: Figure S1A). Moreover, miR-638 overexpression inhibited HCT-116 and SW1116 cell migration by 23.4% and 33.1%, respectively, whereas the inhibition of miR-638 expression enhanced cell migration up to 22.6% and 23.5%, respectively (Figure 2D, Additional file 4: Figure S1B). These data show that miR-638 inhibited CRC cell invasion and migration.

### Loss of miR-638 promotes a mesenchymal-like transition in CRC cells

**Figure 3:**
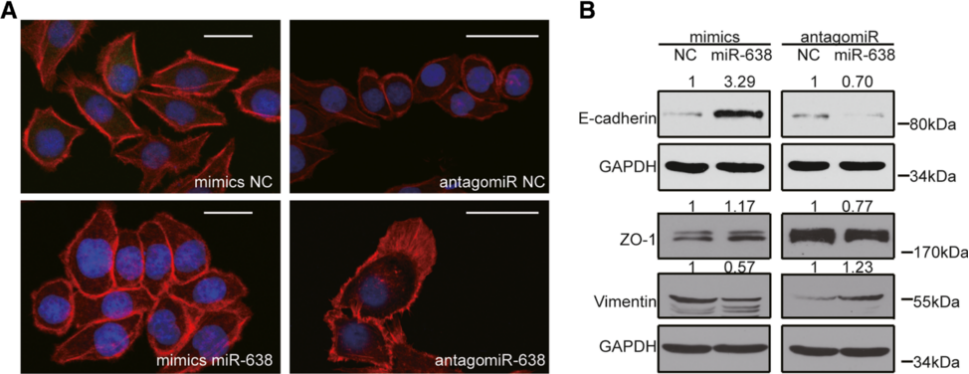
**The inhibition of miR-638 induces a mesenchymal-like transition in SW1116 cells. A)** The overexpression of miR-638 mimics in SW1116 cells increased cell-to-cell contacts, and the overexpression of the antagomiR to miR-638 promoted lamellipodium stretching and decreased cell-to-cell contacts. Cells were stained for F-actin (red). Nuclei were counterstained with DAPI (blue). Scale bar, 20 μm. **B)** The overexpression of miR-638 mimics in SW1116 cells increased the expression of the epithelial cell marker ZO-1/E-cadherin and decreased the expression of the mesenchymal cell marker vimentin. In contrast, the expression of the antagomiR to miR-638 decreased ZO-1/E-cadherin expression and increased vimentin expression.

The loss of miR-638 expression in poorly differentiated CRC tissues and its role in promoting cell migration and invasion suggest that miR-638 may be involved in the EMT process. The mesenchymal-like transition includes changes in cell morphology, markers, and motility. To confirm this hypothesis, we first examined the morphology of SW1116 cells with altered miR-638 expression levels. The data showed that the cell morphology was significantly altered. The cell-to-cell contacts were increased in the cells with miR-638 overexpression; in contrast, when antagomiR-638 was transfected, lamellipodium formation was promoted and cell-to-cell contracts were decreased (Figure 3A). Furthermore, we examined epithelial and mesenchymal cell marker expression levels through Western blotting. In the miR-638 mimic-transfected SW1116 cells, the epithelial cell marker zonula occludens-1 (ZO-1) and E-cadherin were upregulated compared with SW1116 cells transfected with the NC mimics. In contrast, the level of the mesenchymal cell marker vimentin was decreased (Figure 3B). Conversely, in the antagomiR-638-transfected SW1116 cells, ZO-1 and E-cadherin were downregulated compared with the NC antagomiR-transfected SW1116 cells, whereas vimentin levels increased (Figure 3B). Taken together, these results indicate that the inhibition of miR-638 in SW1116 cells resulted in mesenchymal-like features (such as stretched lamellipodia, reduced cell-to-cell contact, decreased epithelial cell marker ZO-1 and E-cadherin expression, and increased mesenchymal cell marker vimentin expression). In contrast, miR-638 overexpression resulted in epithelial-like features (such as increased cell-to-cell contact, increased ZO-1 and E-cadherin expression, and decreased vimentin expression).

### miR-638 directly inhibits SOX2 expression by interacting with its 3′-UTR

**Figure 4:**
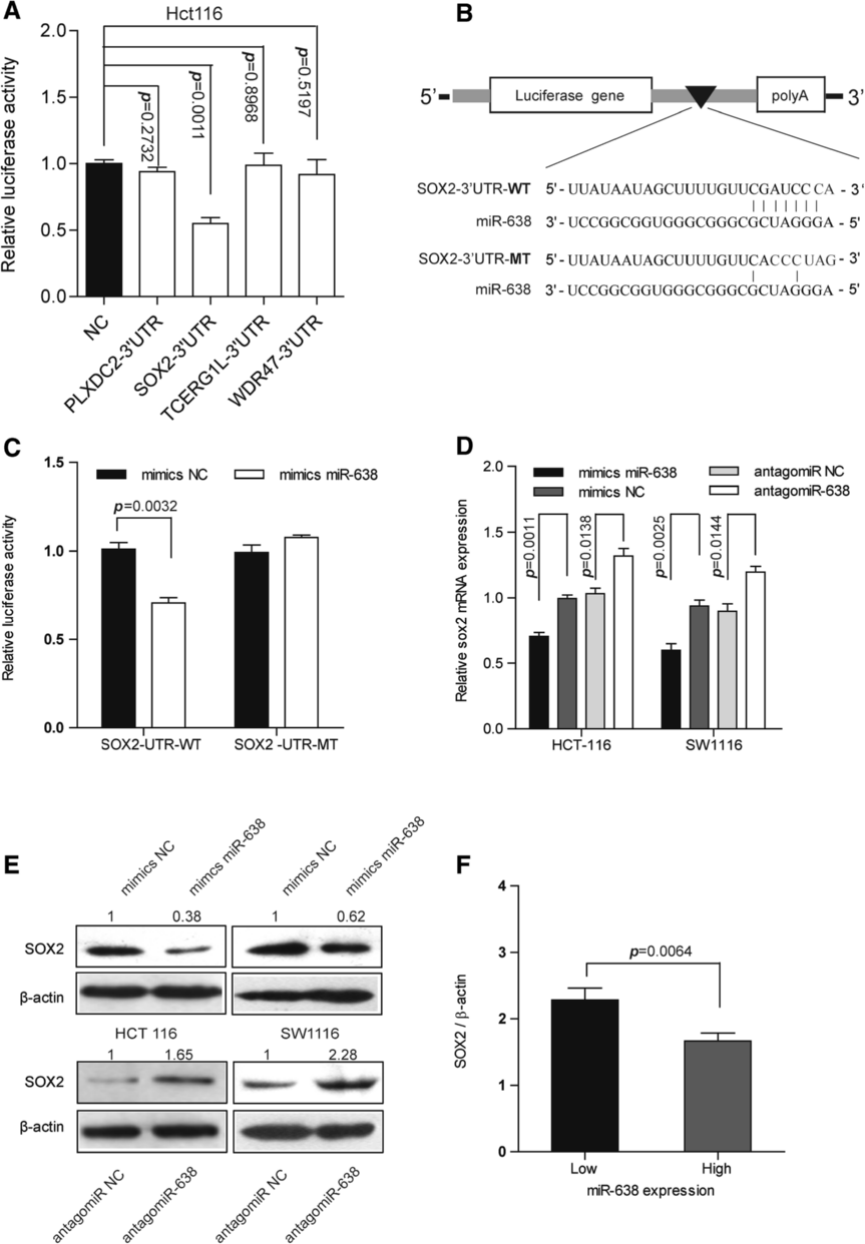
**miR-638 suppresses SOX2 expression by directly targeting its 3′-UTR. A)** The luciferase activity of the luciferase reporter plasmid containing the 3′-UTRs of four putative miR-638 target genes was assessed using the Dual Luciferase Reporter Gene Assay. After transfection with miR-638 mimics for 48 hours, the relative luciferase activity of the plasmid containing the SOX2 3′UTR was inhibited by 45% by miR-638, and the relative luciferase activity of other plasmids exhibited no significant change. Each sample was compared to the negative control (empty vector), and cotransfection of a Renilla plasmid served as an internal control. **B)** A potential miR-638 binding site and the mutated sequence in the SOX2 3′UTR for the seed region are shown. **C)** The luciferase activity of the reporter vector containing either the wild-type (WT) or mutant (MT) SOX2 3′-UTR was assessed after cells were transfected with miR-638 mimics; the mutant abolished the repression by transfection with the miR-638 mimic. **(D)** Changes in miR-638 expression levels drove endogenous SOX2 mRNA expression changes inversely. **(E)** miR-638 expression changes drove SOX2 endogenous SOX2 protein expression changes inversely. **F)** miR-638 expression was inversely correlated with SOX2 mRNA expression in CRC tissues. Based on the miR-638 expression levels in the CRC tissue samples, we sorted these 36 samples into low- and high-expression groups (unpaired *t*-test).

Identification of the target genes of miR-638 is essential for the elucidation of its biological functions. First, we predicted the target genes of miR-638 from miRNA.org, and the top genes with the lowest mirSVR score (cutoff = -1.2, Additional file 5: Table S4) included PLXDC2, SOX2, TCERG1L, and WDR4, which were considered putative candidate targets. Second, the 3′-UTRs of these four genes were subcloned into a luciferase reporter vector to evaluate the influence of miR-638 on the expression of these genes through a luciferase assay (the PCR primers are listed in Additional file 1: Table S5). The results showed that miR-638 more drastically suppressed the luciferase activity of the SOX2 3’-UTR-containing reporter gene construct than the other three constructs (Figure 4A). To further determine whether SOX2 was a *bona fide* target of miR-638, we mutated the predicted binding site of miR-638 on the SOX2 3′-UTR (Figure 4B) and found that the mutant SOX2 3**′**-UTR reporter gene completely abolished miR-638-mediated repression (Figure 4C). Next, we determined the SOX2 mRNA and protein expression levels in HCT-116 and SW1116 cells with altered miR-638 expression levels. Our data revealed that miR-638 overexpression inhibited SOX2 mRNA expression by 69.97% and 61.02% compared with the negative control group in the HCT-116 and SW1116 cells, respectively; furthermore, SOX2 mRNA levels were upregulated in antagomiR-638-transfected HCT116 and SW1116 cells (Figure 4D). Moreover, the SOX2 protein expression levels were downregulated in the miR-638 mimic-transfected CRC cell lines compared with the negative control (mimics negative control), and SOX2 expression was upregulated in the antagomiR-transfected CRC cell lines compared with the negative control (antagomiR negative control; Figure 4E). Furthermore, we detected SOX2 mRNA expression levels in CRC tissues in which miR-638 expression was detected. The data show that the SOX2 mRNA expression level in the miR-638 high-expression group was only 73.04% of that observed in the miR-638 low-expression group (Figure 4F). Taken together, these results indicate that miR-638 inhibited SOX2 expression through direct binding to the 3′-UTR of SOX2.

### SOX2 is highly expressed in CRC tissues

**Figure 5:**
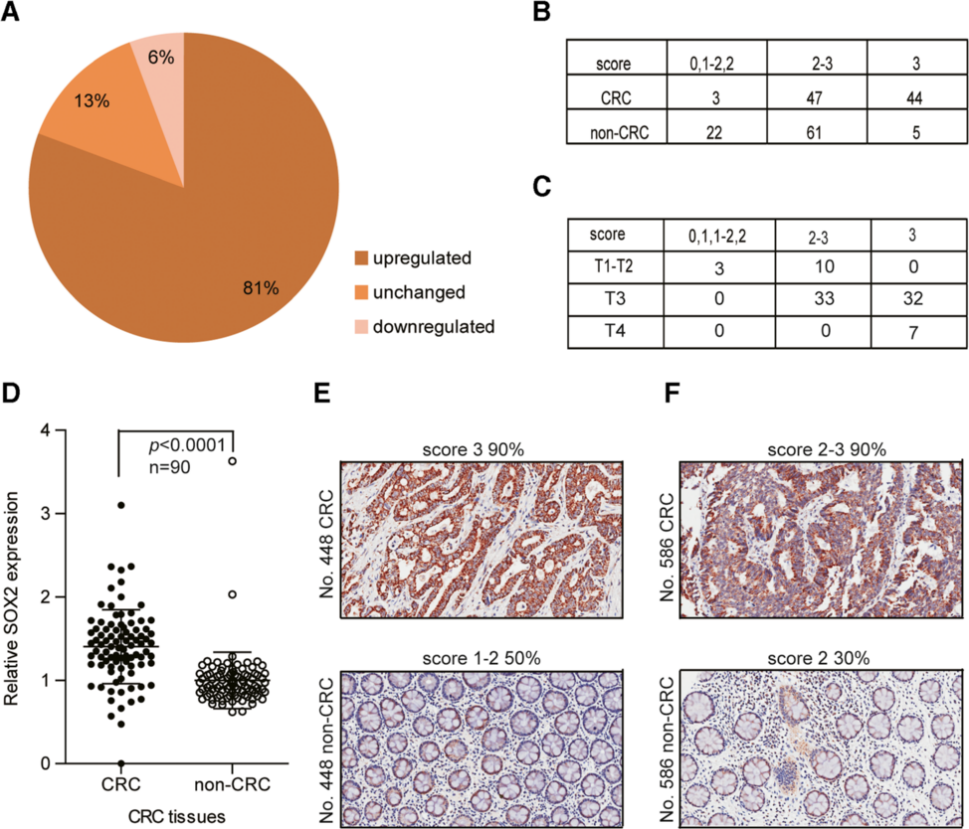
**SOX2 IHC staining intensity is high in CRC tissues. (A)** The staining intensity of SOX2 in the CRC tissue samples was upregulated in 81% of all 90 CRC samples. In addition, the downregulation rate was 6%. The staining intensity was confirmed by a pathologist. **(B)** The staining intensity score was increased in the CRC tissue samples compared with the adjacent, non-cancerous tissue samples (χ^2^ = 47.15, *p* < 0.0001). **C)** The staining intensity score was correlated with the TNM stage of the CRC tissue samples (χ^2^ = 32.53, *p* < 0.0001). **(D)** The SOX2 staining intensity was increased, as assessed using Image-Pro 6.0 software, and the staining intensity was upregulated by 40.45% in the HCC tissues compared with the adjacent non-cancerous tissue samples. **E)** and **F)** A representative image illustrating the upregulation of the SOX2 staining intensity is shown. IHC was performed on 90 pairs of HCC tissue arrays.

To further evaluate the correlation between miR-638 and SOX2 expression in human CRC, we assessed SOX2 expression using immunohistochemistry (IHC) in a CRC tissue array (90 pairs of CRC tissues; Additional file 3: Table S2b). Of the 90 cases, 73 tumors exhibited increased SOX2 expression levels compared with patient-matched, adjacent non-cancerous tissues (Figure 5A). The SOX2 staining scores for the CRC tissue samples were higher than those for their adjacent normal tissue samples (Figure 5B). Furthermore, the SOX2 expression levels in the tumor tissues were inversely correlated with TNM stage (Figure 5C). We then quantified the intensity of SOX2 expression using Image-Pro Plus 6.0 software. The results indicated that the SOX2 staining intensity values were upregulated in CRC tissue samples by 40.45% compared with those of the adjacent non-cancerous tissues (1.4045/1; the SOX2 IHC average intensity value in CRC samples was designated as 1) and in 86.67% of the samples (78/90; Figure 5D). Representative SOX2 IHC staining in CRC tissue samples No. 448 and No. 586 is shown (Figure 5E and 5F). These data demonstrate that SOX2 was frequently highly expressed in CRC tissues compared with adjacent non-cancerous tissues and was associated with tumor grade, in contrast to the miR-638 expression levels in CRC.

### Knockdown of SOX2 expression suppresses cellular invasion in CRC cells

**Figure 6:**
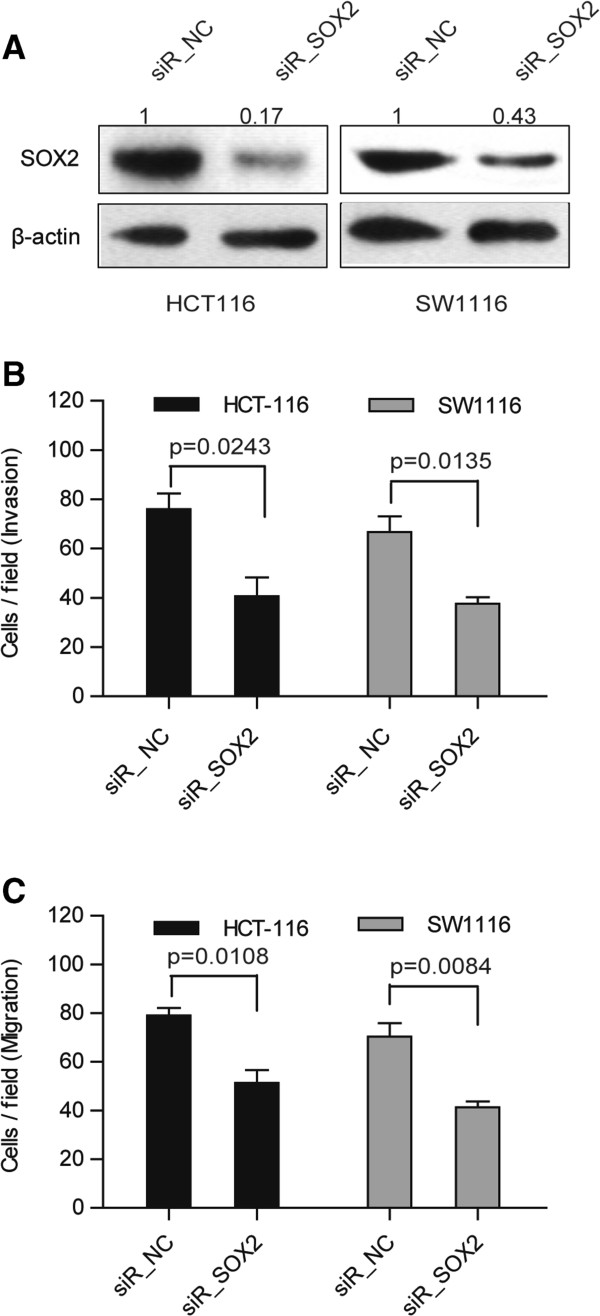
**Knockdown of SOX2 inhibits the cell invasion and migration of CRC cell lines. (A)** SOX2 was knocked down in CRC cells, and its protein expression was verified by Western blotting analysis. Invasion **(B)** and migration **(C)** were inhibited after SOX2 knockdown in HCT-116 and SW1116 cells (unpaired *t*-test). Matrigel-coated (for invasion) and -uncoated (for migration) transwell assays were performed after transfection with SOX2 siRNA for 48 hours.

To further determine whether SOX2 is involved in miR-638-induced CRC cellular invasiveness, we inhibited SOX2 using siRNA, which was confirmed through Western blotting (Figure 6A). The results revealed that SOX2-depleted cells showed significantly reduced cell invasion (Figure 6B, Additional file 6: Figure S2A) and migration (Figure 6C, Additional file 6: Figure S2B), which mimicked the effect of miR-638. These data indicate that the knockdown of SOX2 can suppress CRC cell migration and invasion, similar to miR-638 overexpression.

### SOX2 overexpression rescues the miR-638-induced inhibition of invasion and mesenchymal-like transition

**Figure 7:**
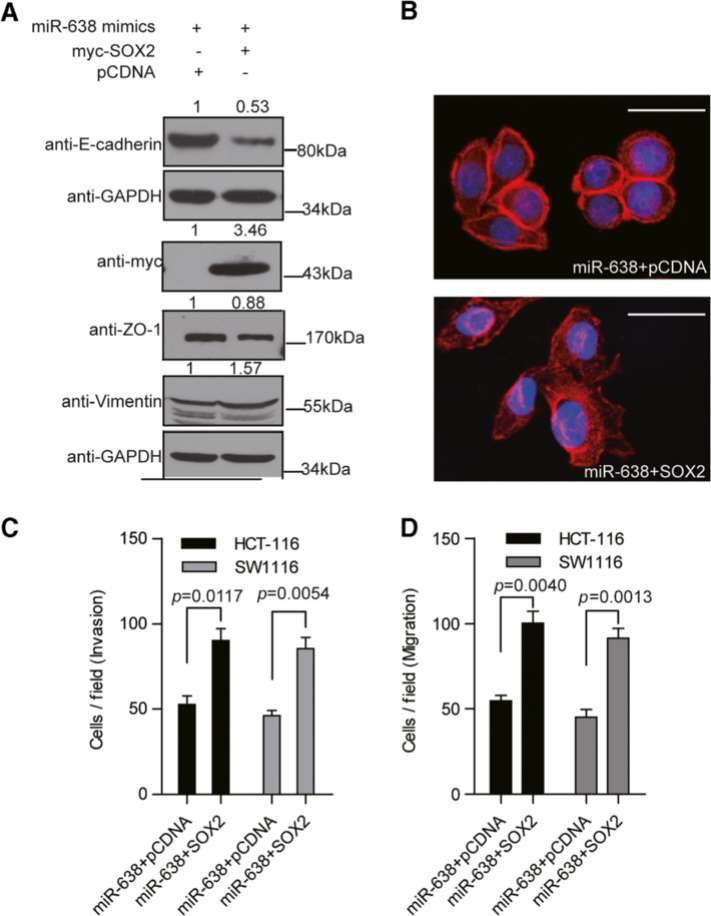
**SOX2 overexpression rescues the miR-638-induced transition to epithelial-like cells. A)** SOX2 overexpression rescued the miR-638-induced transition to epithelial-like cells, as verified by the level of expression of the epithelial cell marker ZO-1/E-cadherin and the mesenchymal cell marker vimentin by Western blotting. SOX2 overexpression was verified by Western blotting using an anti-Myc antibody. SOX2 overexpression in miR-638-transfected SW1116 cells decreased the expression of the epithelial cell marker ZO-1/E-cadherin and increased the expression of the mesenchymal cell marker vimentin. **B)** SOX2 overexpression rescued the miR-638-induced transition to epithelial-like cells, as verified by the cell morphology change. SOX2 overexpression in miR-638-transfected SW1116 cells decreased cell-to-cell contacts and promoted lamellipodium stretching. Cells were stained for F-actin (red). Nuclei were counterstained with DAPI (blue). Scale bar, 20 μm. SOX2 overexpression rescued the miR-638-induced transition to epithelial-like cells, as verified by the cell motility change. The overexpression of SOX2 reversed the miR-638 inhibition of cell invasion **(C)** and cell migration **(D)**.

To determine whether SOX2 is involved in the miR-638-induced inhibition of invasion and mesenchymal-like transition in CRC cells, we increased SOX2 expressions using an overexpression construct in miR-638 mimic-transfected CRC cells. The transfection efficiency was 100% using an Amaxa Nucleofector (with the co-transfection of GFP as an indicator). After the cells were co-transfected with the SOX2 overexpression construct and miR-638, we confirmed SOX2 overexpression by Western blotting and then detected the expression of the epithelial cell marker ZO-1/E-cadherin and the mesenchymal cell marker vimentin. The data showed that the ZO-1/E-cadherin expression levels decreased, whereas the vimentin levels increased (Figure 7A, Additional file 7: Figure S4). Furthermore, the cell morphology changed (Figure 7B), the cell-to-cell contacts decreased, and the lamellipodia were stretched. SOX2 overexpression reversed the miR-638 inhibition of cell invasion and cell migration (Figure 7C and D, Additional file 8: Figure S3).

Taken together, these data indicate that SOX2 can rescue the miR-638-induced inhibition of cell invasion and mesenchymal-like transition.

## Discussion

Tumor metastasis is the major cause of death in CRC patients and occurs in >30% of patients at diagnosis and, subsequently, in >50% of patients after surgery with curative intent [25]. Unfortunately, because of the lack of knowledge regarding the mechanism underlying colorectal tumorigenesis, no effective therapies that block the development and progression of metastasis have been identified. Therefore, the 5-year survival rate of patients with colon cancer is less than 10% if the cancer has metastasized [26,27]. Invasion and a mesenchymal-like transition are the primary processes involved in metastasis. In this study, we aimed to find a new microRNA associated with invasion and the transition to mesenchymal-like cells.

miRNAs are a class of gene expression-regulating molecules that are associated with cancer development and progression. The deregulation of miRNAs is a common event in human cancers, and many miRNAs, such as miRNA-143 [27], miR-9 [28], and miR-137 [29], have been found to be deregulated in CRC and involved in cancer invasion or migration. In a previous study, we found 23 apparently downregulated miRNAs in CRC tissues using a microarray assay [30]. After validating the expression of these miRNAs through qRT-PCR in CRC tissues, we found that miR-638, which has been reported to be downregulated in human gastric cancers [31], basal cell carcinomas [32], and chronic lymphocytic leukemias [33], was obviously downregulated in CRC cancer tissues compared with adjacent tissues. Subsequently, we examined the effect of miR-638 on CRC cell invasion and migration *in vitro* and found that miR-638 particularly inhibited cell invasion and migration, which suggested that miR-638 is an anti-oncomiR and invasion-related miRNA in CRC. Furthermore, miR-638 was correlated with tumor differentiation grade. These data suggest that miR-638 may be involved in the interaction between or conversion of epithelial and mesenchymal cells. Further data showed that miR-638 loss induced a mesenchymal-like transition (such as reduced cell-to-cell contacts, stretched lamellipodia, and upregulation of the mesenchymal cell marker vimentin).

miR-638 plays certain roles in pathology and physiology. miR-638, which was downregulated in non-small-cell lung cancer (NSCLC) tissues, aggravated DNA damage (induced by benzo (a) pyrene) by suppressing breast cancer 1 (BRCA1). Moreover, one study indicated that miR-638 is downregulated at the invasive fronts of colorectal liver metastases based on microarray analysis [16]. In this study, we found that miR-638 exhibited reduced expression in CRC tissues, and this loss of expression promoted cell invasion and a mesenchymal-like transition.

The regulatory functions of miRNAs are mediated by their target genes; therefore, it is essential to identify the specific genes that are targeted by those miRNAs. The mirSVR score that is provided at mircrorna.org ranks microRNA target sites using a downregulation score [34]. This score is used to identify target genes or predict the extent of their downregulation at the mRNA or protein levels [35,36]. Using this method, SOX2 was chosen as a candidate target gene. Using a series of assays (for example, a reporter assay to validate that SOX2 is the only and direct target, miR-638 alterations drove SOX2 expression, miR-638 expression was inversely correlated with SOX2 expression in CRC tissues, SOX2 knockdown phenocopied the overexpression of miR-638, and SOX2 overexpression in miR-638-transfected cells rescued miR-638-induced function), SOX2 was finally identified as the functional target gene of miR-638 in CRC.

SOX2 is a Yamanaka factor that can induce pluripotent stem cells from mouse embryonic and adult fibroblast cultures [17]. The EMT process is accompanied by increased SOX2 expression [37] and can be induced via the SOX2 pathway [38]. The overexpression of SOX2 promotes dedifferentiation and induces the EMT process [39], whereas the knockdown of SOX2 induces the MET process [40,41]. Furthermore, SOX2 expression (by conventional IHC) is correlated with lymph node metastasis; therefore, it can serve as a metastasis marker for CRC [42]. In the present study, we used a more precise assay (ompare to Conventional immunohistochemical expression analysis), IHC in tissue arrays, and found that SOX2 was overexpressed in CRC tissues and was correlated with tumor grade.

In summary, our results suggest that miR-638 is a potential invasion-associated tumor suppressor in CRC. In this study, we confirmed that miR-638 exhibited reduced expression in CRC, and its expression was correlated with tumor differentiation grade. Furthermore, the inhibition of miR-638 induced CRC cell lines to develop mesenchymal-like cell features (e.g., reduced cell-cell contact, increased lamellipodium stretching, decreased expression of the epithelial cell marker ZO-1/E-cadherin, increased expression of the mesenchymal cell marker vimentin, and increased cell migration and invasion), and we confirmed that SOX2 is a direct target of miR-638. These findings may facilitate the development of new CRC therapeutics.

## Competing interests

The authors declare that they have no competing interests.

## Authors’ contributions

XL and KM designed and performed the experiments and discussed and interpreted the data. KM, XP, PF, YH, JG, WW, and ZT performed the experiments. ZL gave suggestions on study design and discussed and interpreted the data. XYL designed and supervised the study, discussed and interpreted the data, and wrote the manuscript. All authors read and approved the final manuscript.

## Supplementary Material

Additional file 1 Tables (S1a, S2a, S3, S5). Click here for file

Additional file 2 Supplementary material and methods: Staining was analyzed based on the percentage of positively stained cells and staining intensity by a pathologist or using Image-Pro Plus 6.0 software. Click here for file

Additional file 3: Table S1b and S2b Summary of Clinicopathologic Variables. Click here for file

Additional file 4: Figure S1 Representative figures for cell migration and invasion in miR-638 mimic- and antagomiR-638-transfected cells. Cell migration and invasion were examined by Matrigel-coated (for invasion) and Matrigel-uncoated (for migration) transwell assays after transfection with miR-638 mimics or antagomiR-638 for 24 h. Representative figures for invasion (A) and migration (B) are shown. Click here for file

Additional file 5: Table S4 miR-638 Prediction targets. Click here for file

Additional file 6: Figure S2 The representative figures of cell migration and invasion in siRNA NC- and siR SOX2- transfected cells. Invasion (A) and migration (B) were examined after transfection with siRNA NC and siR SOX2 in CRC cells for 48 h. Click here for file

Additional file 7: Figure S4 The raw material of Western Blot in Figure 7A. Click here for file

Additional file 8: Figure S3 The representative figures of cell migration and invasion in miR-638 mimic- and SOX-overexpressing cells. Invasion (A) and migration (B) were examined after transfection with miR-638 mimics and pCDNA_SOX2 in CRC cells for 48 h. Click here for file
