# Supplementary material for: Loss of miR-638 in vitro promotes cell invasion and a mesenchymal-like transition by influencing SOX2 expression in colorectal carcinoma cells
Source: Mol Cancer. 2014 May 23;13:118. doi: 10.1186/1476-4598-13-118 (PMC4039649; doi:10.1186/1476-4598-13-118)
Supplement: Additional file 1 — Tables (S1a, S2a, S3, S5). [file 1476-4598-13-118-S1.doc]

| **Supplementary Table S1a. Summary of Clinicopathologic Variables** | | |
| --- | --- | --- |
| Characteristic | Number of Patients |  |
| Patients | 36 |  |
| Gender |  |  |
| male | 20 |  |
| female | 16 |  |
| Age (years) | 32-78, median=61.5 |  |
| TNM stage |  |  |
| I | 1 |  |
| II | 13 |  |
| III | 20 |  |
| IV | 2 |  |
| T stage |  |  |
| T1 | 1 |  |
| T2 | 6 |  |
| T3 | 15 |  |
| T4 | 14 |  |
| Differentiation |  |  |
| Low | 4 |  |
| Median | 23 |  |
| High | 10 |  |
| Histology |  |  |
| adenocarcinoma | 32 |  |
| mucinous adenocarcinoma | 4 |  |
| signet ring cell cancer | 0 |  |
| Location |  |  |
| Rectum | 21 |  |
| Proximal colon | 7 |  |
| Distal colon | 8 |  |

**Supplementary Table S2a**. Summary of Clinicopathologic Variables

| Characteristic | Number of Patients |
| --- | --- |
| Patients | 90 |
| Gender |  |
| male | 60 |
| female | 30 |
| Age (years) | 30-91, median=65 |
| TNM stage |  |
| I | 4 |
| I-II | 6 |
| II | 56 |
| II-III | 13 |
| III | 11 |
| Tumor size (cm) |  |
| < 5 | 77 |
| >5 | 13 |
| Histology |  |
| mucinous adenocarcinoma | 4 |
| Tubular adenocarcinoma | 33 |
| signet ring cell cancer | 1 |
| adenocarcinoma | 52 |
| Time of follow-up (months) | 1-73, median=61 |

**Supplementary** Table S3. Significant downregulation of miRNAs in primary CRC

| miRNA Name | Fold change(NCT/T) * | P value# |
| --- | --- | --- |
| hsa-miR-29c* | 2.04 | <0.01 |
| hsa-miR-145* | 2.13 | <0.01 |
| hsa-miR-1826 | 2.13 | <0.01 |
| hsa-miR-638 | 2.22 | <0.01 |
| hsa-miR-497 | 2.22 | <0.001 |
| hsa-miR-572 | 2.33 | <0.01 |
| hsa-miR-195 | 2.38 | <0.001 |
| hsa-miR-326 | 2.44 | <0.01 |
| hsa-miR-338-3p | 2.50 | <0.001 |
| hsa-miR-378 | 2.50 | <0.001 |
| hsa-miR-1 | 2.63 | <0.01 |
| hsa-miR-145 | 2.63 | <0.01 |
| hsa-miR-133b | 2.78 | <0.01 |
| hsa-miR-375 | 2.86 | <0.01 |
| hsa-miR-513c | 3.03 | <0.01 |
| hsa-miR-650 | 3.57 | <0.01 |
| hsa-miR-381 | 4.00 | <0.01 |
| hsa-miR-378* | 4.00 | <0.001 |
| hsa-miR-551b | 4.76 | <0.001 |
| hsa-miR-9 | 7.69 | <0.001 |
| hsa-miR-30a* | 9.09 | <0.001 |
| hsa-miR-139-5p | 10.00 | <0.001 |
| hsa-miR-204 | 12.50 | <0.001 |

*: T: tumor tissue; NCT: noncancerous tissue

#:P-values are calculated on log-signal of microRNA expression measured by microarray.

**Supplementary** Table S5. Sequences of PCR primers

| Primers | Sequence (5 'to 3')* | Products length |
| --- | --- | --- |
| PLXDC2-UTR-F | ATGGAATTCGTTTCATATACACTGGAGAAGTC | 506bp |
| PLXDC2-UTR-R | CACTCTAGAGCCAATCTGAGAAGATACAC |  |
| SOX2-UTR-F | ATG GAATTC ATTTTTCAAGGAGAGGCTTC | 472bp |
| SOX2-UTR-R | CACTCTAGA TACATGGATTCTCGGCAGAC |  |
| TCERG1L-UTR-F | ATG GAATTC CACGGTGGAGACGGACAC | 496bp |
| TCERG1L-UTR-R | CAC TCTAGA CCACCCTTGGAAACCTGTAA |  |
| WDR47-UTR-F | ATG GAATTC GAGCACACCGCATGTCAGTC | 769bp |
| WDR47-UTR-R | CAC TCTAGA TGCTGCTGCTCTGCTTCTAC |  |

* Sequences underlined are sites for restriction enzymes.
