# Supplementary material for: Loss of miR-638 in vitro promotes cell invasion and a mesenchymal-like transition by influencing SOX2 expression in colorectal carcinoma cells
Source: Mol Cancer. 2014 May 23;13:118. doi: 10.1186/1476-4598-13-118 (PMC4039649; doi:10.1186/1476-4598-13-118)
Supplement: Additional file 2 — Supplementary material and methods: Staining was analyzed based on the percentage of positively stained cells and staining intensity by a pathologist or using Image-Pro Plus 6.0 software. [file 1476-4598-13-118-S2.doc]

Staining was analysed based on the percentage of positively stained cells and staining intensity by pathologist who did not know the aim of the research.

1, Location decision: cytoplasm, membrane or nucleus

2, qualitative judgment: negative or positive

3, Quantitative judgment

A, the staining intensity: At low magnification of the view point was observed, which is divided into weak positive, positive and strong positive. The weakly positive light yellow (+ or 1), the positive Brown (++ or 2), strong positive Brown (+++ or 3). (Note: if the organization has positive and strong positive we usually denoted as 2-3, similar results as above; the immunohistochemical experiments using DAB color)

B, the positive rate of staining: First at low magnification to observe the tissue of the field, and then select 3 staining intensity of different view of interpretation at high magnification, if it is localized in the nucleus, we are in each field were recorded 100 cells, then write 100 cells in the positive cells accounted for the percentage of X^1^%, the percentage of positive cells in the same principle 2 vision after accounting for X^2^%, X^3^%, the average number of the tissue staining positive rate of (X^1^% + X^2^% + X^3^%)/3; if located in the cytoplasm or cell membrane, we choose 3 different staining intensities were estimated its vision, the average positive rate.

Staining was analysed based on the percentage of positively stained cells and staining intensity by the Image-Pro Plus 6.0 software as describely in Hepatology. 2012 Jun;55(6):1787-98. doi: 10.1002/hep.25596.
