# Supplementary figures and images for: Loss of miR-638 in vitro promotes cell invasion and a mesenchymal-like transition by influencing SOX2 expression in colorectal carcinoma cells
Source: Mol Cancer. 2014 May 23;13:118. doi: 10.1186/1476-4598-13-118 (PMC4039649; doi:10.1186/1476-4598-13-118)

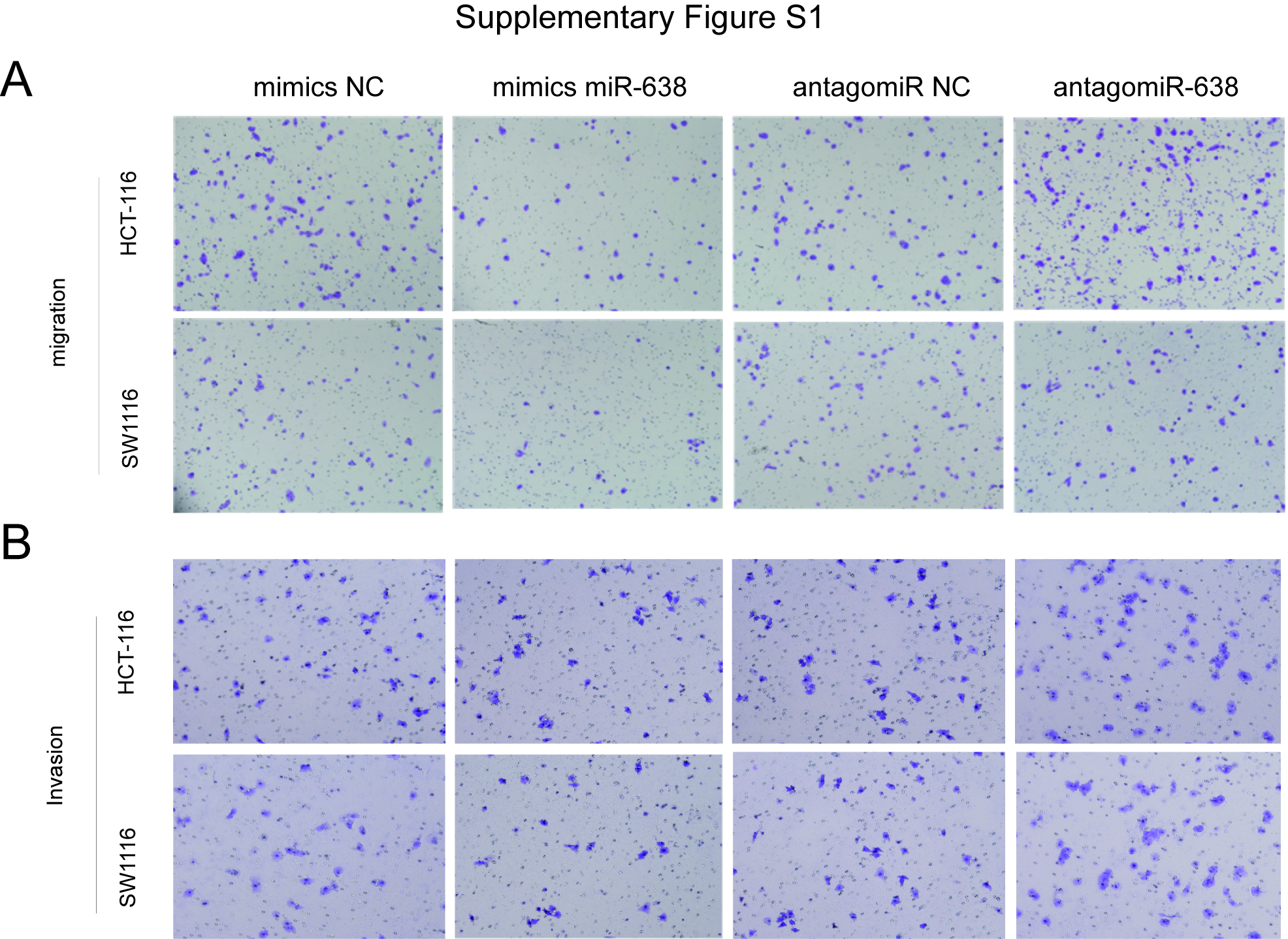

Supplement: Additional file 4: Figure S1 — Representative figures for cell migration and invasion in miR-638 mimic- and antagomiR-638-transfected cells. Cell migration and invasion were examined by Matrigel-coated (for invasion) and Matrigel-uncoated (for migration) transwell assays after transfection with miR-638 mimics or antagomiR-638 for 24 h. Representative figures for invasion (A) and migration (B) are shown. [file 1476-4598-13-118-S4.tiff]

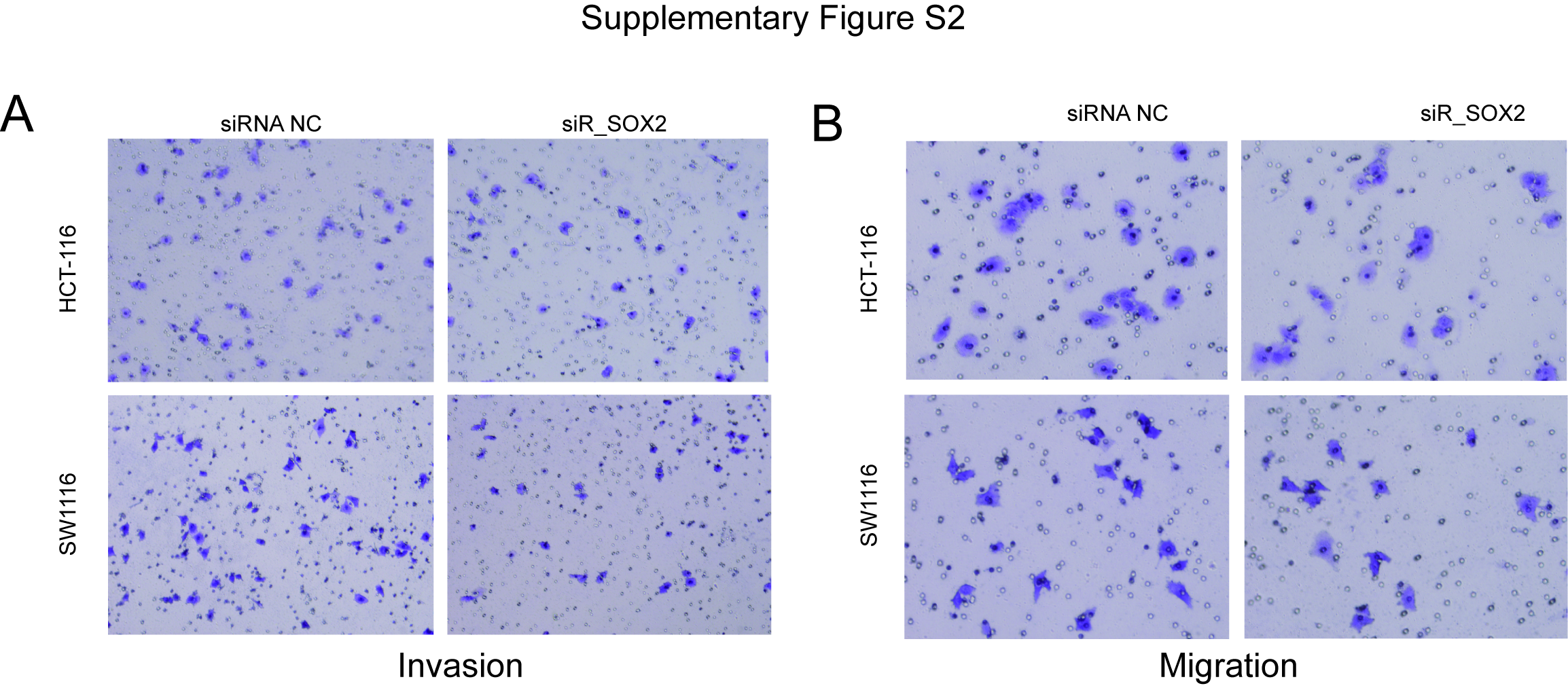

Supplement: Additional file 6: Figure S2 — The representative figures of cell migration and invasion in siRNA NC- and siR SOX2- transfected cells. Invasion (A) and migration (B) were examined after transfection with siRNA NC and siR SOX2 in CRC cells for 48 h. [file 1476-4598-13-118-S6.tiff]

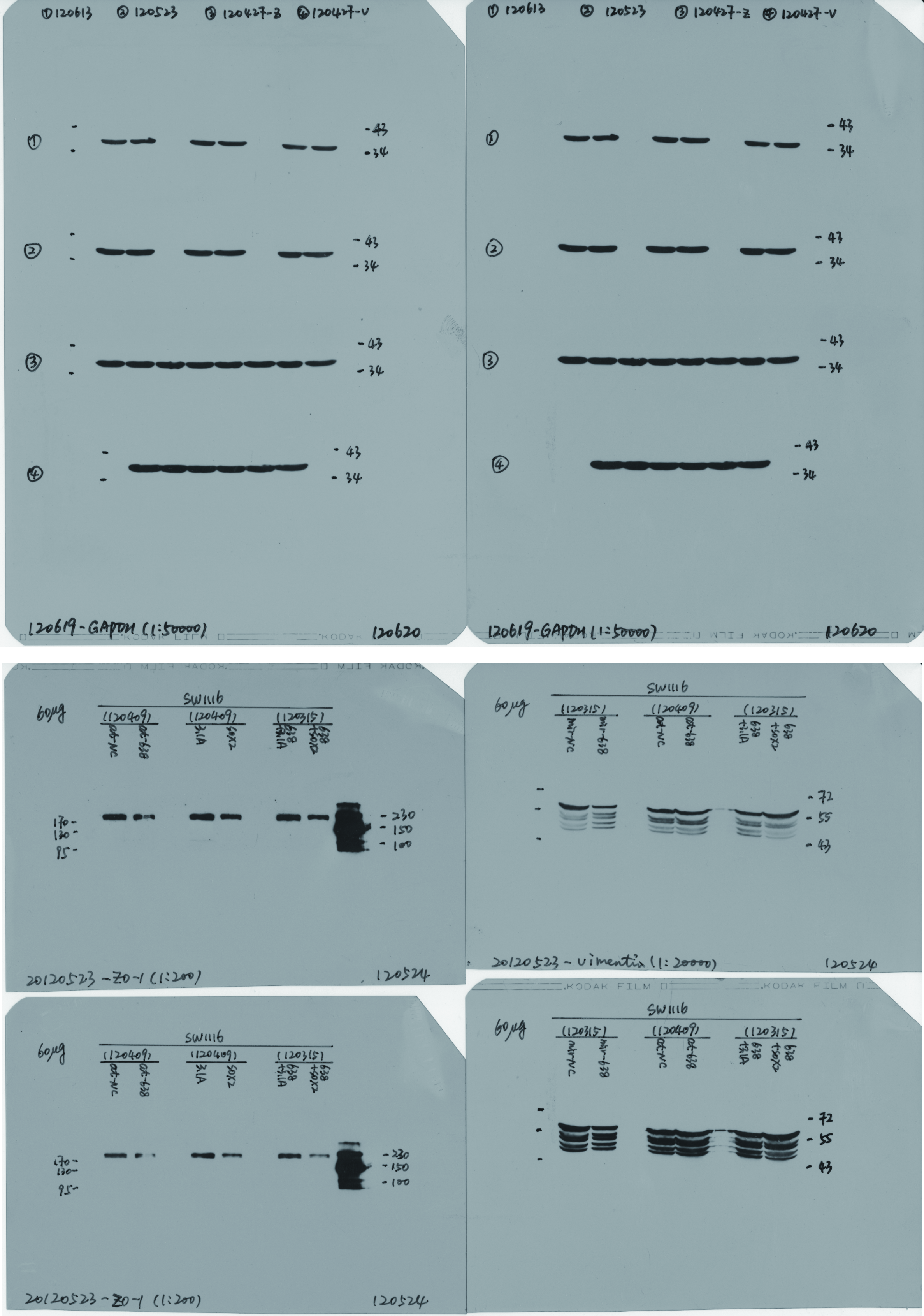

Supplement: Additional file 7: Figure S4 — The raw material of Western Blot in Figure 7A. [file 1476-4598-13-118-S7.tiff]

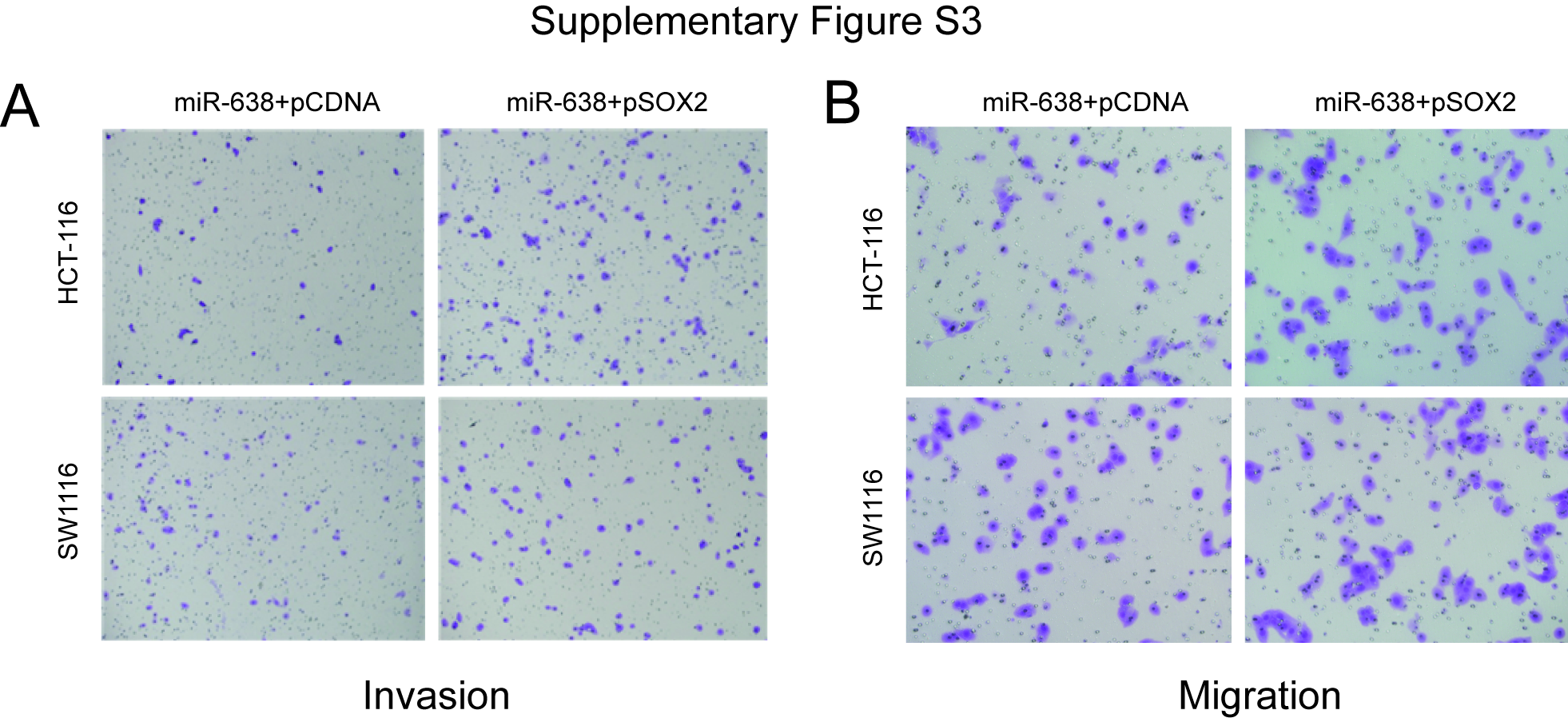

Supplement: Additional file 8: Figure S3 — The representative figures of cell migration and invasion in miR-638 mimic- and SOX-overexpressing cells. Invasion (A) and migration (B) were examined after transfection with miR-638 mimics and pCDNA_SOX2 in CRC cells for 48 h. [file 1476-4598-13-118-S8.tiff]
