# Supplementary material for: Loss of miR-638 in vitro promotes cell invasion and a mesenchymal-like transition by influencing SOX2 expression in colorectal carcinoma cells
Source: Mol Cancer. 2014 May 23;13:118. doi: 10.1186/1476-4598-13-118 (PMC4039649; doi:10.1186/1476-4598-13-118)
Supplement: Additional file 5: Table S4 — miR-638 Prediction targets. [file 1476-4598-13-118-S5.docx]

| Gene_symbol | miRNA_alignment | Alignment | Gene_alignment | Conservation | miRsvr_score |
| --- | --- | --- | --- | --- | --- |
| PLXDC2 | uccGGCGGUGGGCG---GGCGCUAGGGa | \|\| ::\| : \|\| :: \|\|\|\|\|\|\| | gcaCCUUUAAUGGCAAAUUUCGAUCCCa | 0.8184 | -1.3098 |
| SOX2 | uccggcggUGGGCGGGCGCUAGGGa | :\|:: : : \|\|\|\|\|\|\| | uuauaauaGCUUUUGUUCGAUCCCa | 0.6662 | -1.2934 |
| TCERG1L | uccGGCGGUGGGCGGGCGCUAGGGa | \|\|\|: \|: \|\|: \|\|\|\|\|\|\| | ucaCCGUGAUACGU---CGAUCCCu | 0.7754 | -1.2791 |
| WDR47 | uccGGCGGUG-GGCGGGCGCUAGGGa | \|: :::\| \|: \|:\| \|\|\|\|\|\|\| | uuaCUUUUGCUCUUCUCACGAUCCCa | 0.5862 | -1.1972 |

**Supplementary Table S4 miR-638 Prediction targets**
